# Characterization of a pathogenic gain-of-function mutation in the N-terminal domain of STAT1 which is reported to be associated with eosinophilic esophagitis

**DOI:** 10.1186/s12964-025-02330-9

**Published:** 2025-08-07

**Authors:** Kristin Annawald, Anke Gregus, Oliver Wirths, Thomas Meyer

**Affiliations:** 1 Department of Psychosomatic Medicine and Psychotherapy, University Medical Centre Göttingen, and German Centre for Cardiovascular Research (DZHK), Göttingen, Germany; 2 Department of Psychiatry and Psychotherapy, University Medical Centre, Göttingen, Germany

**Keywords:** STAT1, N-terminal domain, Gain-of-function, Eosinophilic esophagitis, Tyrosine phosphorylation

## Abstract

The pathophysiology of eosinophilic esophagitis (EoE), a chronic allergic disease characterized by eosinophilic infiltration of the esophageal mucosa, is largely unknown. Recently, a case report described a gain-of-function (GOF) mutation in the STAT1 (signal transducer and activator of transcription 1) protein (D65A) to be associated with this disease. In the present paper, we investigated in more detail the molecular mechanisms of this missense mutation and, in addition, characterized a second aspartic acid-to-alanine substitution (D66A) in the N-terminal domain of STAT1. Results showed that, upon stimulation of cells with cytokines, the two mutants had increased levels of tyrosine phosphorylation compared to the wild-type (WT) protein. The altered phosphorylation kinetics led to an elevated and prolonged phase of nuclear accumulation, which was in line with an increased concentration of DNA-bound complexes observed by means of electrophoretic mobility shift assays. However, the dissociation rate from a single high-affinity DNA-binding site did not differ between the WT and the two mutants. A promoter-specific higher transcriptional activation was observed for reporter gene constructs and the majority of the tested endogenous STAT1 target genes. In summary, the two N-terminal point mutations showed characteristic features of a GOF phenotype, as indicated by a gene-specific, rather than a global upregulation of cytokine-driven gene expression. A model is proposed suggesting that the equilibrium between antiparallel, tetrameric complexes and parallel dimer complexes is shifted to the transcriptionally active latter ones as the underlying mechanistic basis of these N-terminal STAT1 mutations.

**Supplementary Information:**

The online version contains supplementary material available at 10.1186/s12964-025-02330-9.

## Introduction

Eosinophilic esophagitis (EoE) is a chronic relapsing, allergic disease marked by histological evidence of eosinophilic infiltration into the esophageal mucosa [1, 2]. Its clinical manifestations vary with age, encompassing symptoms such as dysphagia and abdominal pain [3, 4]. Adolescent and adult patients frequently present with retrosternal chest pain, dysphagia, odynophagia and spontaneous food impaction, whereas children may exhibit more unspecific symptoms, like nausea, vomiting, reflux, regurgitation, food refusal, abdominal pain and even failure to thrive [3]. Current diagnostic criteria of EoE involve typical endoscopic findings in the affected esophagus, including edema, exudates, circular rings, linear furrows, plaques, and/or strictures, and a mandatory histopathological evidence of at least 15 eosinophilic granulocytes per high-power field (HPF) in biopsies.

Pathophysiologically, the condition appears to be triggered by exposure to allergens leading to persistent accumulation of inflammatory T-helper cells (Th2) located in the esophageal mucosa with subsequent tissue damage and consecutive remodeling [5, 6]. The etiology is multifactorial as epigenetic and environmental factors play a major role in the development of EoE [2]. Several genetic risk loci have been linked to EoE including the genes *CAPN14*, *TSLP* and *STAT6*, which are involved in epithelial homeostasis, tissue repair, Th2 cell development and interleukin (IL) signaling [7]. Twin studies have demonstrated a weak genetic heritability of the disease with phenotypic variation [8].

Scott and colleagues (2022) reported a case study of a 39-year-old woman and two female first-degree relatives aged 5 and 70 years, who were affected by chronic mucocutaneous candidiasis (CMC) [9]. Notably, both adults had persistent functional oesophageal dysfunction with dysphagia and episodes of food impaction, but without evidence of esophageal fungal infection. Based on histopathological examination, EoE was diagnosed in the index patient. Using genomic sequencing, a heterozygous missense mutation (c.194 A > C, p.D65A) was found in the gene encoding STAT1 (signal transducer and activator of transcription 1), which resulted in a single amino-acid exchange in the N-terminal domain.

**Fig. 1:**
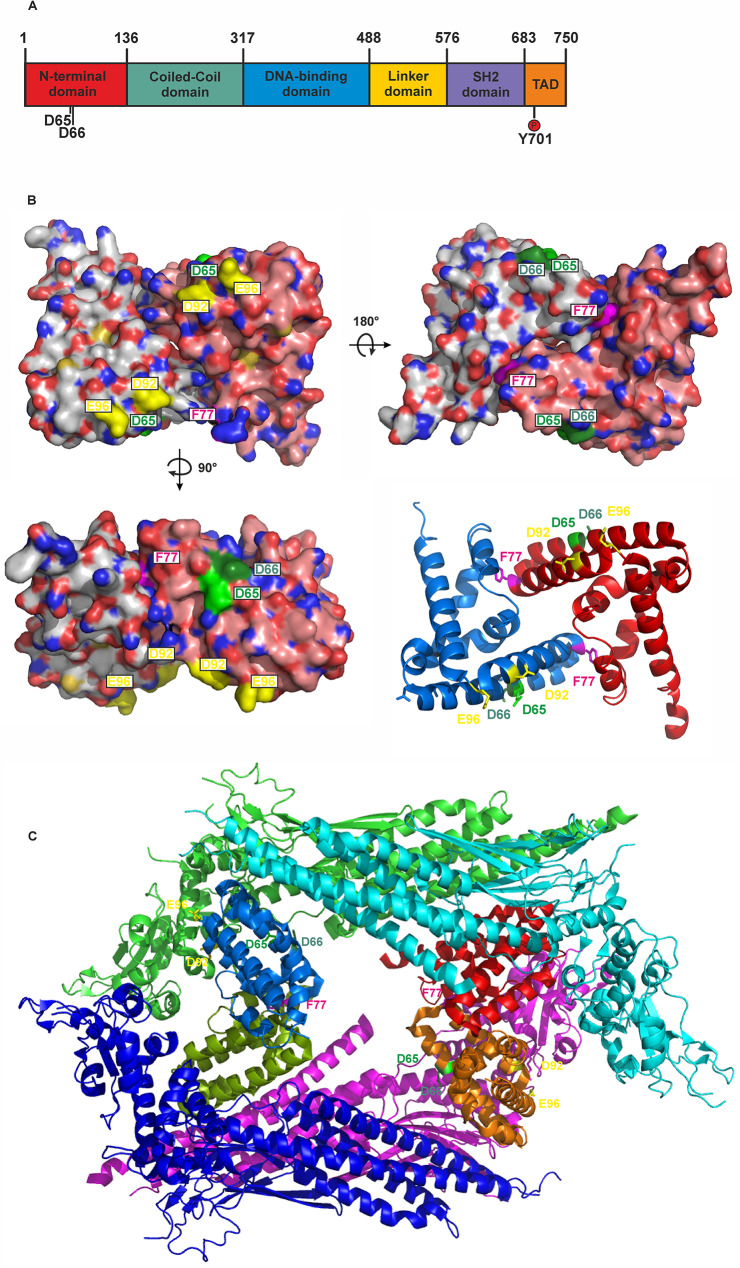
Localization of two N-terminal STAT1 gain-of-function mutations. **(A)** Position of two N-terminal GOF mutations D65 and D66 in the domain structure of STAT1 including the critical tyrosine residue Y701 in the transactivation domain (TAD). **(B)** Surface structure of the N-terminal dimer of STAT1 in orthogonal views and the ribbon diagram thereof. Shown is the localization of crucial residues involved in N-terminal interactions: F77 (magenta), D92 and E96 (both yellow) and the amino-acid residues under investigation D65 (light green) and D66 (dark green). The two monomers are shown in different colors. **(C)** Ribbon diagram of the crystal structure of the antiparallel tetramer including the two aspartic acid residues. The four N-terminal domains in the interior complex as well as the truncated core proteins are colored differently. Protein crystal structure renderings from the Protein Data Bank file 1YVL [32] were generated using the PyMOL software (DeLano Scientific)

STAT1 belongs to a protein family of cytokine-driven transcription factors, which are involved in various cellular functions such as differentiation, inflammation, growth and apoptosis [10]. All family members share a similar domain architecture consisting of six domains: the N-terminal (ND), coiled-coil (CCD), DNA-binding (DBD), linker (LD), Src homology 2 (SH2) and the transactivation (TAD) domain (Fig. 1A) [11]. Following cytokine binding to specific cell surface receptors, STAT1 undergoes phosphorylation at the single tyrosine residue 701 by receptor-associated Janus-activated kinases (JAKs). This phosphorylation step enables the formation of parallel dimers through reciprocal interactions between the phosphotyrosine 701 residue and the SH2 domain of the partner protomer [11, 12]. Upon activation of the JAK/STAT pathway by interferon-γ (IFNγ), tyrosine-phosphorylated STAT1 homodimers are transported into the nucleus by importin-α5 [13, 14]. Inside the nucleus, the dimers bind to γ-activated sites, termed GAS elements, on DNA to modulate the transcription of interferon-stimulated genes, which play an essential role in regulating innate immune responses [15].

Gain-of-function (GOF) mutations in the *STAT1* gene have been described as a cause of an altered immune response against invading pathogens. Frequently, pathogenic STAT1 GOF mutations associated with CMC are located in the coiled-coil (amino acid positions 136–316) and DNA-binding (317–487) domain [16, 17]. Using a mutagenesis approach, the study presented aims to explore the molecular mechanisms behind the N-terminal GOF substitution mutation D65A linked to EoE.

## Materials and methods

### Plasmids, mutagenesis and cell culture

All experiments were performed using HeLa cells or STAT1-deficient U3A cells [18, 19, 20]. Cells were maintained at 37 °C in a humidified atmosphere containing 5% CO₂. HeLa cells were cultured in Roswell Park Memorial Institute (RPMI) 1640 medium (Anprotec, with stable glutamine) supplemented with 10% fetal bovine serum (FBS) (Biochrom) and 100 IU/ml penicillin/streptomycin. U3A cells were grown in Dulbecco’s modified Eagle’s medium (DMEM) (Anprotech, high glucose (4.5 g/L), with stable glutamine) containing 10% FBS, 100 IU/ml penicillin/streptomycin, and 0.04 µg/ml puromycin (Sigma-Aldrich). Transfection was carried out using MegaTran2.0 (Origene) and, on the following day, cells were either left untreated or stimulated with 50 ng/ml of recombinant human IFNγ (Biomol) for the specified durations. For transfection of HeLa and U3A cells, the following expression vectors were used in this study: pEGFPN1-STAT1α [21], pSTAT1α-Flag [22, 23], pcDNA3.1-STAT1α [14] and the construct pSTAT1α-NES-GFP [24]. The pEGFPN1-STAT1α vector encodes a fusion protein comprising full-length human, wild-type STAT1 (WT-GFP, amino acids 1–746) and green fluorescent protein (GFP), while pSTAT1α-Flag is a vector encoding Flag-tagged STAT1-WT [22]. The plasmid pcDNA3.1-STAT1α codes for full-length human untagged STAT1-WT. For fluorescence microscopy, we used the construct pSTAT1α-NES-GFP, which contains a sequence coding for a STAT1-derived artificial nuclear export signal (NES) obtained from amino acids 367–427 located between the cDNAs for full-length STAT1 and GFP [24, 25]. The above-mentioned plasmids served as templates for introducing point mutations through site-directed mutagenesis, performed with the QuikChange II kit (Stratagene) according to the manufacturer’s protocol. The primers used for mutagenesis were prepared at a concentration of 125 ng/µl, with the forward strands listed below (mutated codons are marked).

D65Af; 5’– GACCTCCTGTCACAGCTG***GCT***GATCAATATAGTCGC–3’, and.

D66Af; 5’– CCTGTCACAGCTGGAT***GCT***CAATATAGTCGCTTTTC–3’.

All point mutations were confirmed using standard Sanger DNA sequencing (Seqlab).

### Protein extraction and Western blotting 

Cells grown in 6-well plates expressing GFP-tagged, Flag-tagged or untagged recombinant STAT1 were lysed in 70 µl of cytoplasmic extraction buffer (20 mM HEPES, pH 7.4, 10 mM KCl, 10% (v/v) glycerol, 1 mM EDTA, 0.1 mM Na₃VO₄, 0.1% IGEPAL-CA-360, 3 mM DTT, 0.4 mM Pefabloc (Sigma-Aldrich), and Complete Mini protease inhibitors (Roche)) on ice for 5 min. Cellular extracts were subjected to centrifugation at 16,000 g for 30 s at 4 °C, and the supernatants were again centrifuged under the same conditions for 5 min. The supernatants of this second centrifugation step were then collected as cytoplasmic extracts. The pellets obtained from the first centrifugation step were resuspended in 70 µl of nuclear extraction buffer (20 mM HEPES, pH 7.4, 420 mM KCl, 20% (v/v) glycerol, 1 mM EDTA, 0.1 mM Na₃VO₄, 3 mM DTT, 0.4 mM Pefabloc, and Complete Mini protease inhibitors) and incubated on ice for 30 min. The samples were centrifuged at 16,000 g for 15 min at 4 °C, and 40 µl of the nuclear extracts were combined with an equal volume of cytoplasmic extracts from the corresponding sample to generate whole-cell lysates. The cellular extracts were denatured in sodium dodecyl sulfate (SDS) sample buffer at 95 °C for 3 min, separated by 10% SDS-polyacrylamide gel electrophoresis (SDS-PAGE), and transferred onto polyvinylidene fluoride (PVDF) membranes. Membranes were blocked with 4% bovine serum albumin (BSA) in Tris-buffered saline supplemented with 0.05% Tween-20 (TBS-T) for 1 h, followed by overnight incubation at 4 °C with the respective primary antibody. The following primary antibodies were used, all diluted in 1:1000 in blocking solution (4% BSA in TBS-T): rabbit monoclonal antibodies against phospho-Tyr701-STAT1 (Cell Signaling Technology, 58D6), pan-STAT1 (Cell Signaling Technology, D1K9Y), and the mouse monoclonal anti-Flag antibody (Sigma Aldrich, M2). After thorough washing, the blots were incubated with conjugated anti-rabbit (IRDye 800CW) or anti-mouse (IRDye 680LT) secondary antibodies (LI-COR Biosciences), diluted 1:1000 in 4% BSA/TBS-T. The bound immunoreactivity was visualized using the LI-COR Odyssey imaging system.

### Immunofluorescence microscopy

To assess the phosphorylation status of the GFP- and Flag-tagged STAT1 variants, U3A and HeLa cells cultured on 8-well Lab-Tek chamber slides (Nunc) were transfected with the respective expression plasmids. On the next day, the cells were stimulated with IFNγ (50 ng/ml) for 45 min, and subsequently treated with the kinase inhibitor staurosporine (1 µM) for the indicated durations. Cells were fixed with cold methanol at -20 °C for 15 min and then permeabilized with 1% Triton X-100 in phosphate-buffered saline (PBS) for 20 min at room temperature (RT) under constant shaking, and then washed thrice with PBS. To block non-specific antibody binding, cells were incubated with 25% FBS in PBS for 45 min at RT while being shaken. Samples were then exposed to phospho-STAT1 58D6 or anti-Flag M2 monoclonal antibodies, both diluted 1:1000 in 25% FBS-PBS for 45 min at RT under constant, gentle shaking. For the detection of bound immunoreactivity, cells were washed three times with PBS and then incubated for 45 min at RT with either Cy3-conjugated anti-rabbit secondary antibody from goat or a Cy3-conjugated anti-mouse secondary antibody from donkey (Jackson ImmunoResearch), diluted 1:1000 in 25% FBS-PBS. Fixed cells were washed twice with PBS and nuclei were counterstained for 10 min with 5 µg/ml of Hoechst dye 33258 (Sigma-Aldrich), followed by additional washes. Samples were mounted in fluorescence mounting medium (Southern Biotech), and the intracellular fluorescence localization was visualized using a Nikon Eclipse Ti fluorescence microscope with appropriate filters. Images were captured with a Nikon DS-Qi2 camera and processed using NIS Elements software (Nikon). Fluorescence intensities in the nucleus and cytoplasm were quantified using ImageJ (NIH), and the mean nuclear-to-total fluorescence intensities, including standard deviations, were calculated from at least 15 transfected cells.

### Electrophoretic mobility shift assay

Electrophoretic mobility shift assays (EMSAs) were used to assess the DNA-binding activity of the STAT1 variants to duplex oligonucleotides containing either single or tandem GAS sites. Extracts from STAT1-expressing and IFNγ-pretreated cells were loaded on a 4.8% non-denaturing acrylamide:bisacrylamide (29:1) gel at 4 °C and STAT1-DNA complexes were separated by means of electrophoresis at 400 V. [³³P]-labeled duplex oligonucleotides were prepared through an end-filling reaction using Klenow fragment polymerase (New England Biolabs). The probes contained either a single canonical GAS binding site (M67) or two GAS elements in tandem orientation, in which no (2xGAS), one (GAS-non-GAS) or both GAS (2xnon-GAS) elements were mutated. The sequences of the DNA probes were as follows (GAS motifs are marked; only forward strands are shown):

M67; 5’-CGACAT***TTCCCGTAA***ATCTG-3′,

2xGAS; 5’-CGT***TTCCCCGAA***ATTGACGGAT***TTCCCCGAA***AC-3′,

GAS-non-GAS; 5‘-CGT***TTCCCCGAA***ATTGACGGATTTACCCCAAC-3‘, and.

2xnon-GAS; 5’-CGTTTACCCCAAATTGACGGATTTACCCCAAC-3’.

For each reaction, 4.5 µl of whole-cell extracts were incubated with 8.5 µl of reaction buffer containing 1 ng of the above-mentioned duplex oligonucleotide probe. For competition assays, samples from mutant STAT1-expressing cells were diluted 1:2 with an equal volume of lysates from non-transfected U3A cells to ensure similar loading of phosphoproteins per lane. The cell extracts were then incubated with [³³P]-labeled duplex oligonucleotides in EMSA reaction buffer for 30 min on ice, followed by the addition of a 750-fold molar excess of unlabeled M67 DNA at the indicated time points. DNA-binding activity was visualized on vacuum-dried gels using the Typhoon FLA 9500 laser phosphor-imaging system (GE Healthcare Life Sciences).

### Reporter gene assay

Differential gene expression was analysed using reporter gene assays in transfected U3A cells expressing GFP-tagged WT and mutant STAT1. To this end, cells were cultured in 48-well plates and co-transfected with 250 ng of the respective STAT1 expression plasmid, 70 ng of a luciferase reporter plasmid, and 200 ng of a constitutively expressed β-galactosidase plasmid. Three luciferase-based reporter constructs were used: 3xLy6E, pIC-339, and pIC-1352. The 3xLy6E construct contains three copies of the IFNγ-inducible Ly6E GAS element located upstream of the transcription start site in the lymphocyte antigen 6 complex locus E promoter region [26]. The pIC-1352 construct comprises the full-length promoter region of the human intercellular adhesion molecule 1 (*ICAM-1*) gene, while pIC-339 is a truncated version thereof with a length of 339 bp [27]. One day post-transfectionem, cells were either left untreated or stimulated with IFNγ for 6 h, before cellular proteins were extracted using a lysis buffer containing 25 mM glycylglycine, 1% Triton X-100, 15 mM MgSO₄, 4 mM EGTA, 0.4 mM Pefabloc, 3 mM DTT (pH 7.8), and Complete protease inhibitors. Luciferase activity was measured using the luciferase assay substrate solution (Promega) on a Tecan Spark multimode microplate reader (Tecan Group) and normalized to β-galactosidase activity, which was quantified spectroscopically at 420 nm. The experiment was performed in triplicate using 6 independent transfections for each combination of STAT1 variants and stimulation modes.

### Quantitative PCR

To evaluate the expression of endogenous STAT1 target genes, transfected U3A cells expressing untagged STAT1-WT, -D65A or -D66A were starved in DMEM supplemented with 1% FBS for 15 h. Cells were then either left untreated or stimulated with 50 ng/ml of IFNγ for 6 h. Total cellular RNA was extracted using the peqGold Total RNA kit (VWR Life Sciences), and first-strand cDNA synthesis was performed using the Verso cDNA Synthesis kit (Thermo Fisher Scientific). Real-time PCR reactions were conducted in a final volume of 20 µl, consisting of 25 ng of cDNA, 70 nM of each specific primer, and 10 µl of Absolute Blue qPCR SYBR Green Mix (Thermo Fisher Scientific). The following primer pairs were used:

GAPDHf 5′-GAA GGT GAA GGT CGG AGT C-′3,

GAPDHr 5′-GAA GAT GGT GAT GGG ATT TC-′3,

STAT1f 5′-CCG TTT TCA TGA CCT CCT GT-′3,

STAT1r 5′-TGA ATA TTC CCC GAC TGA GC-′3,

IRF1f 5′-AGC TCA GCT GTG CGA GTG TA-′3,

IRF1r 5′-TAG CTG CTG TGG TCA TCA GG-′3,

MCP1f 5′-CCA GTC ACC TGC TGT TAT AAC-’3,

MCP1r 5′-TGG AAT CCT GAA CCC ACT TCT-′3,

GBP1f 5′-GGT CCA GTT GCT GAA AGA GC-′3,

GBP1r 5′-TGA CAG GAA GGC TCT GGT CT-′3,

MIGf 5′-CCA CCG AGA TCC TTA TCG AA-′3,

MIGr 5′-CTA ACC GAC TTG GCT GCT TC-′3,

CXCL10f 5′-ATT CTG AGC CTA CAG CAG AG-′3, and.

CXCL10r 5′-GCT TGC AGG AAT AAT TTC AA-′3.

The PCR protocol, executed on an Eppendorf cycler, included an initial denaturation step at 95 °C for 15 min, followed by 40 cycles of denaturation at 95 °C for 15 s, annealing at 55 °C for 30 s, and extension at 72 °C for 30 s. After the final amplification step, a melting curve analysis was performed using a temperature gradient from 60 °C to 95 °C in 0.5 °C increments, with fluorescence being measured at each temperature for a period of 10 s. All reactions were carried out in at least triplicate independent experiments. The relative transcript expression levels were normalized to GAPDH expression for each sample. The ΔΔCt method based on the formula 2^−(ΔCt target − ΔCt reference sample)^ was used to calculate relative expression levels.

### Statistical analysis

Digital images were processed using ImageJ software (NIH), and data figures were generated with CorelDRAW Graphics Suite 2021. Statistical analyses were carried out using GraphPad Prism 10 for Windows. Differences between WT and mutant STAT1 variants were assessed using Student’s *t*-test with Welch’s correction for statistical significance. Results are expressed as the mean ± standard deviation of the mean with a significance level set at *p* ≤ 0.05. Significant differences are indicated by asterisks in the corresponding graphs.

## Results

### Two aspartic acid residues in the N-terminal domain modulate STAT1 tyrosine phosphorylation

**Fig. 2:**
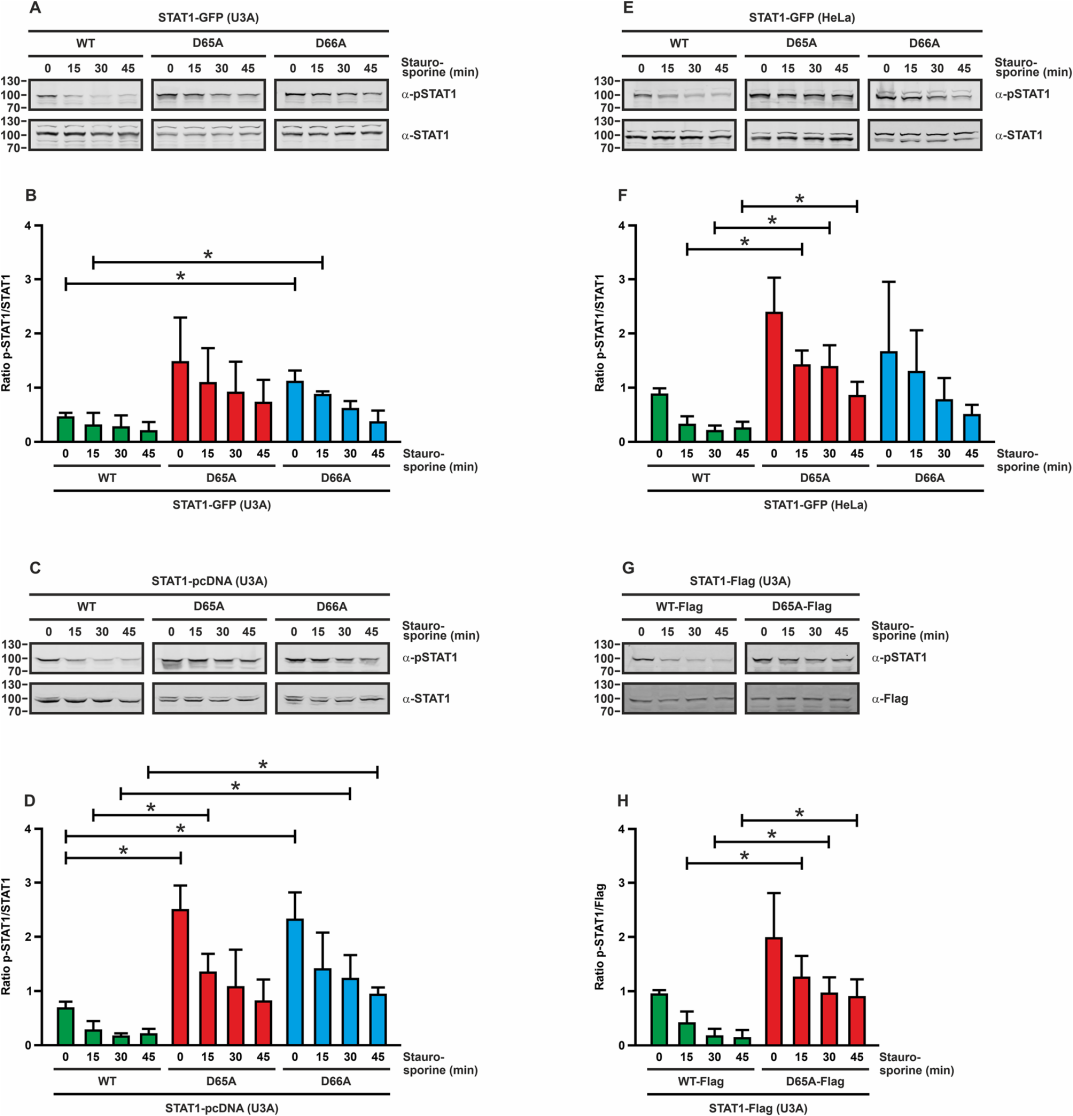
Hyperphosphorylation of the STAT1-D65A and -D66A mutants. Western blot results from whole-cell extracts using U3A cells **(A–D**,** G**, **H)** expressing GFP-tagged **(A**,** B)**, untagged **(C**,** D)** or Flag-tagged **(G**,** H)** STAT1 proteins and HeLa cells expressing STAT1-GFP **(E**,** F)**. Cells expressing either WT or mutant STAT1- D65A and -D66A were stimulated with 50 ng/ml of IFNγ for 45 min and then treated with 1 µM of the kinase inhibitor staurosporine for the indicated times. Immunoblots were probed with a phosphotyrosine-specific STAT1 antibody (α-pSTAT1) to assess STAT1 phosphorylation and a pan-STAT1 (α-STAT1) or Flag antibody (α-Flag) to detect total STAT1 protein levels. The quantifications of the blots from at least three independent experiments are shown in **(B**,** D**, **F**,** H)**. Statistically significant differences between WT protein and mutant STAT1 are indicated by asterisks

In this project, two aspartic acid residues in the N-terminal domain, namely D65 and D66, were mutated to alanine and the resulting missense mutants were analysed for their kinetics of cytokine-induced tyrosine phosphorylation by means of immunoblotting. The two amino-acid residues are located in close proximity with their functional carboxyl groups pointing to the surface of the domain (Fig. 1). Mutations were introduced in plasmids encoding untagged, GFP-tagged or Flag-tagged STAT1 and the resulting variants were expressed in transfected HeLa or STAT1-reconstituted U3A cells. Following IFNγ stimulation of the transfected HeLa or U3A cells, the two mutants showed increased STAT1 tyrosine phosphorylation levels and, moreover, displayed prolonged duration of tyrosine phosphorylation in the presence of the kinase inhibitor staurosporine. The elevated and prolonged phase of tyrosine phosphorylation was seen independently of whether untagged STAT1 or recombinant fusion proteins with GFP- or Flag-tags were studied (Fig. 2).

### Enhanced transcriptional activity by STAT1-D65A and -D66A

**Fig. 3:**
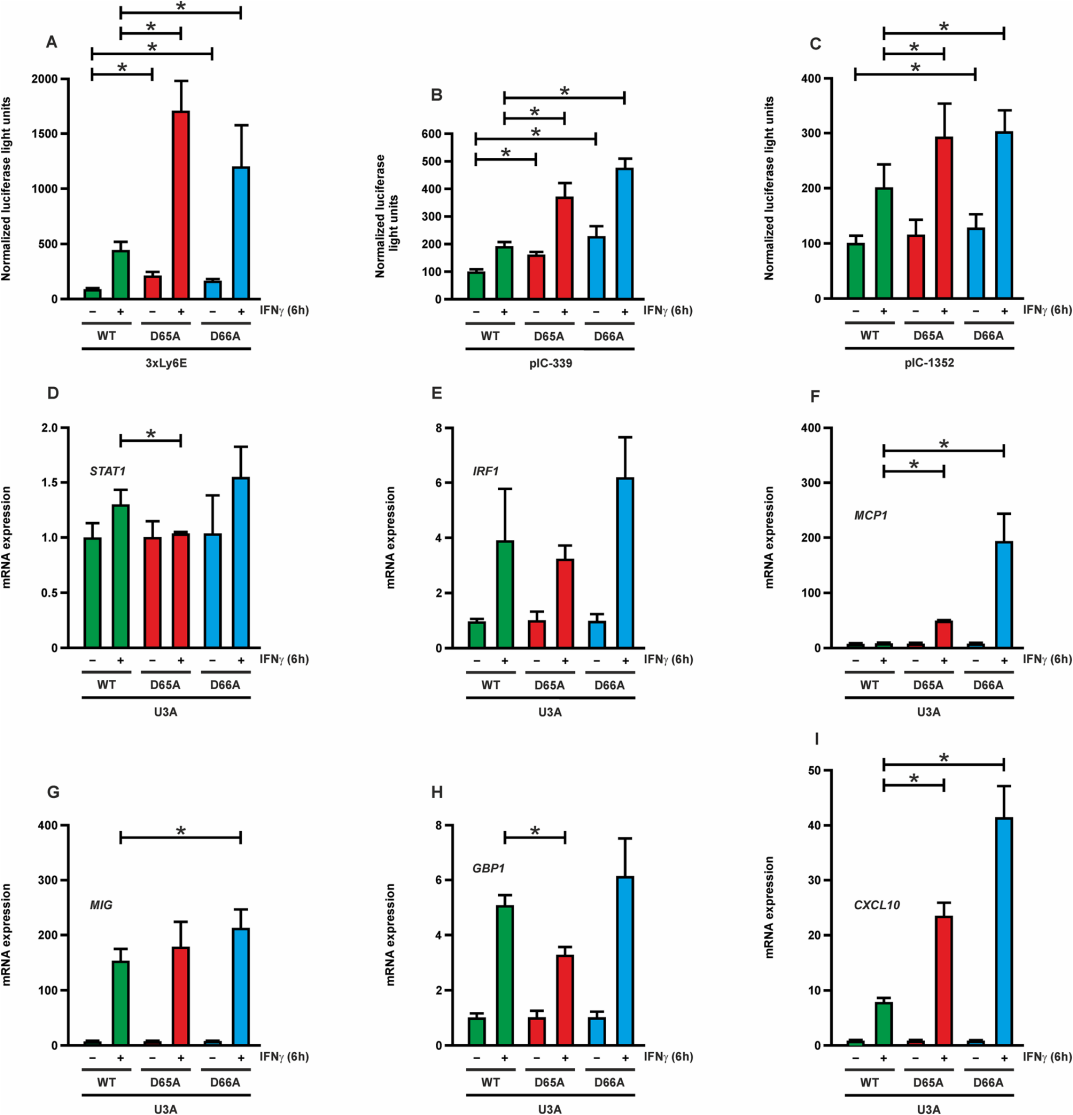
Increased gene-specific transcriptional activity of D65A and D66A. **(A–C)** STAT1-negative U3A cells were transfected with expression plasmids coding for GFP-tagged fusion proteins of WT, D65A or D66A variants in combination with a luciferase reporter gene construct (3xLy6E, pIC-339, or pIC-1352) and a constitutively expressed β-galactosidase plasmid used for normalization. Cells were stimulated with 50 ng/ml of IFNγ for 0 h (-) or 6 h (+) before extracts were tested for luciferase and β-galactosidase production. Significant differences between the WT and mutant proteins are marked with asterisks. **(D–I)** Real-time PCR analysis was performed to compare mRNA production of endogenous STAT1 target genes for WT and the tested mutants. Cells were treated with 50 ng/ml of IFNγ for 6 h (+) or left untreated (-). The following gene transcripts were examined: *STAT1***(D)**, *IRF1***(E)**, *MCP1***(F)**, *MIG***(G)**, *GBP1***(H)**, and *CXCL10***(I)**. Significant differences between WT and the mutants are highlighted by asterisks

To assess whether the two hyperphosphorylated STAT1 mutants D65A and D66A differ from the WT molecule with respect to gene expression, we firstly evaluated the generated mutants for their reporter gene activity using a luciferase assay. A significantly increased luciferase light signal was observed for all three different promoter constructs, namely an artificial promoter containing three high-affinity GAS binding sites (3xLy6E) as well as the endogenous *ICAM-1* pIC-339 and pIC-1352 promoter regions (Fig. 3A–C). Using real-time PCR assays, the three STAT1 variants showed an elevated level of gene expression for a number of known *STAT1* endogenous target genes, when the reconstituted cells were pre-treated with IFNγ. In particular, the *MCP1* and *CXCL10* genes were highly upregulated by the two substitution mutants, since their promoters contained a one-and-a-half consensus GAS binding site (Fig. 3D–I). However, the D65A mutant did not act as a better transcriptional activator than the WT protein on all IFNγ-inducible target genes.

### The D65A and D66A mutants showed normal dissociation rates from DNA

**Fig. 4:**
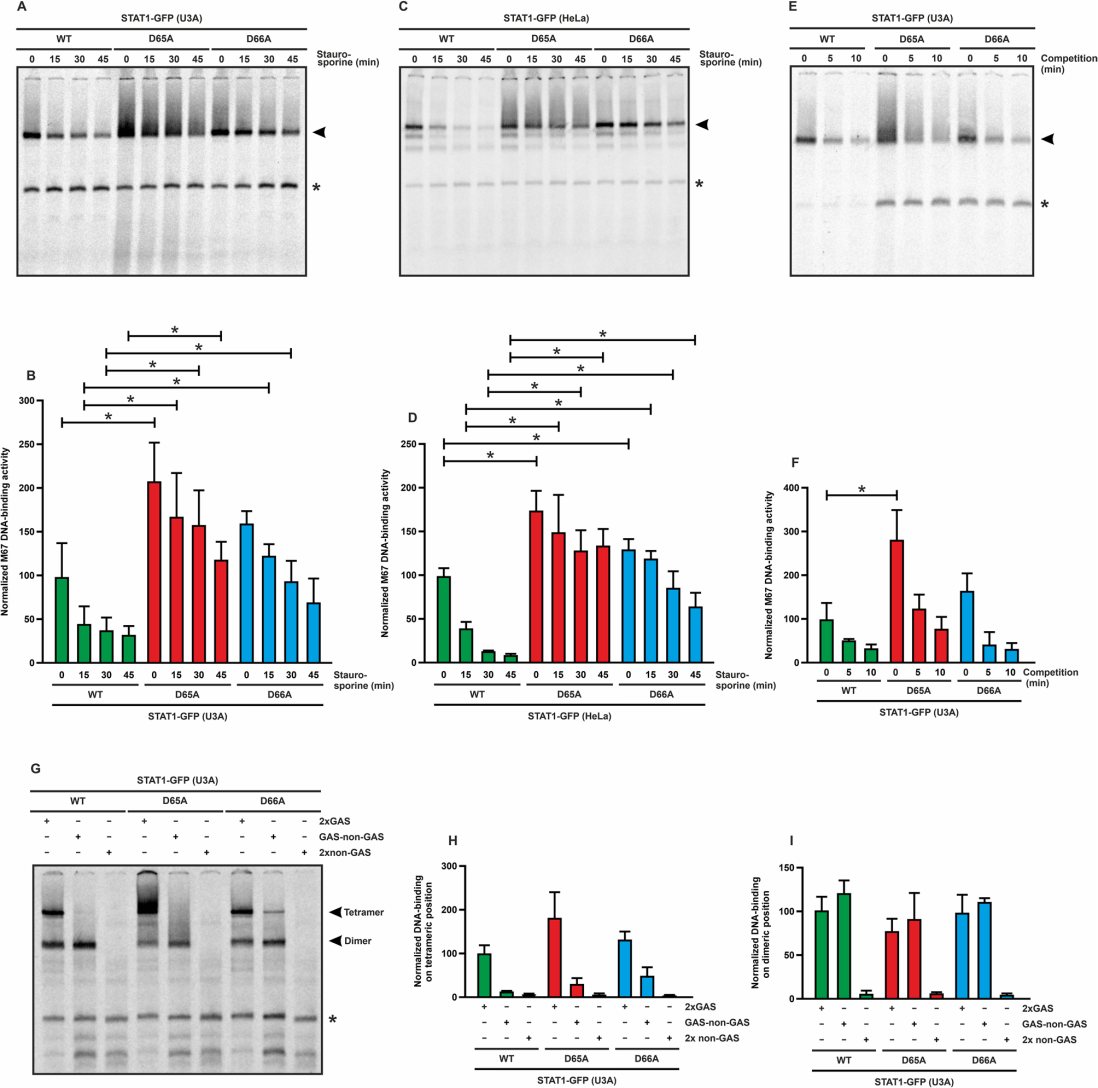
Normal DNA-binding affinity of two N-terminal GOF mutants. **(A–D)** Kinetics of DNA binding to a [^33^P]-labeled, single GAS site (M67) using extracts from reconstituted U3A cells **(A**,** B)** or HeLa cells **(C**,** D)** expressing either WT, D65A or D66A. Cells were stimulated with 50 ng/ml IFNγ for 45 min and subsequently treated with 1 µM staurosporine for the indicated times. Extracts were mixed with the M67 probe for 5 min before being separated by electrophoresis. Representative autoradiograms **(A**,** C)** including the quantification of three experiments **(B**,** D)** are shown. Note that the increased DNA-binding intensity of the mutants compared to WT reflects their elevated level of tyrosine phosphorylation, as demonstrated from the Western blot data in Fig. 2. Arrowheads indicate binding on DNA as dimers, and unspecific bands are marked with an asterisk. Asterisks in all quantification graphs represent significant differences observed between the WT protein and its respective mutants. **(E)** Representative autoradiogram from a competition gelshift experiment using U3A whole cell extracts expressing the indicated STAT1 variants and a 750-fold excess of unlabeled M67 as competitor. To ensure equal loading of phospho-proteins, extracts from mutant-expressing cells were diluted 1:2 with extracts from non-transfected U3A cells. The [^33^P]-labeled M67 probe was incubated with the samples for 30 min, followed by exposure to the unlabeled M67 for increasing time intervals (0 min, 5 min, and 10 min) on ice. Arrowhead indicates binding on DNA as dimers, and an unspecific band is marked with an asterisk. **(F)** Quantification of EMSA competition results from three experiments as shown in **(E)**. Note the similar dissociation kinetics of the WT protein and the mutants. **(G–I)** Similar sequence-specific DNA binding of D65A and D66A compared to the WT molecule. Shown is a representative autoradiogram of a gelshift experiment using STAT1-reconstituted cells and different DNA probes with increasing affinity containing no GAS site (2xnon-GAS), one single GAS site (GAS-nonGAS) or two GAS sites in tandem orientation (2xGAS). Transfected cells expressing the indicated STAT1 variants were stimulated for 45 min with IFNγ, before the corresponding extracts were incubated for 5 min at RT with the [^33^P]-labeled DNA probes. The different bands for tetramers and dimers are highlighted and an unspecific band is marked with an asterisk. **(H**,** I)** Quantification of normalized DNA-binding affinity for the three different tandem GAS elements on the tetrameric **(H)** and dimeric **(I)** position

The kinetics of DNA binding for the STAT1 mutants under investigation were tested in gelshift assays using [^33^P]-labeled double-stranded oligonucleotides. To this end, transfected U3A or HeLa cells expressing the recombinant STAT1 protein variants were treated as described above, before their cell lysates were incubated with the DNA probe containing a single GAS site (M67) and subsequently loaded on a non-denaturing polyacrylamide gel. The autoradiograms showed the prolonged and elevated DNA binding of the tyrosine-phosphorylated STAT1 mutants as compared to the WT protein, confirming the results from the Western blots experiments. The stronger DNA-binding activity of the mutants resulting from their hyperphosphorylation was associated with a smear-like effect particularly seen for D65A, suggesting partial denaturation of higher molecular-weight structures (Fig. 4A–D).

To examine the dissociation rate from DNA, a competition gelshift experiment was performed in the presence of 750-fold molar excess of unlabeled M67 for different intervals. Results showed that there were no significant differences between the WT protein and the two mutants. This finding indicated that the dissociation rate of dimeric STAT1 from a single GAS element did not differ among the three variants (Fig. 4E, F).

Sequence specificity of DNA binding was tested using a double GAS site aligned in tandem orientation, termed 2xGAS, compared to permutated sequences thereof with one (GAS-non-GAS) or no (2xnon-GAS) preserved GAS site. All three mutants showed the same preference for sequence-specific DNA binding favouring the 2xGAS element over the permutated forms. The tetrameric band observed for binding of D66A to GAS-non-GAS most likely resulted from the elevated tyrosine phosphorylation level of this mutant (Fig. 4G–I).

### The mutants exhibit prolonged nuclear accumulation as phospho-proteins

**Fig. 5:**
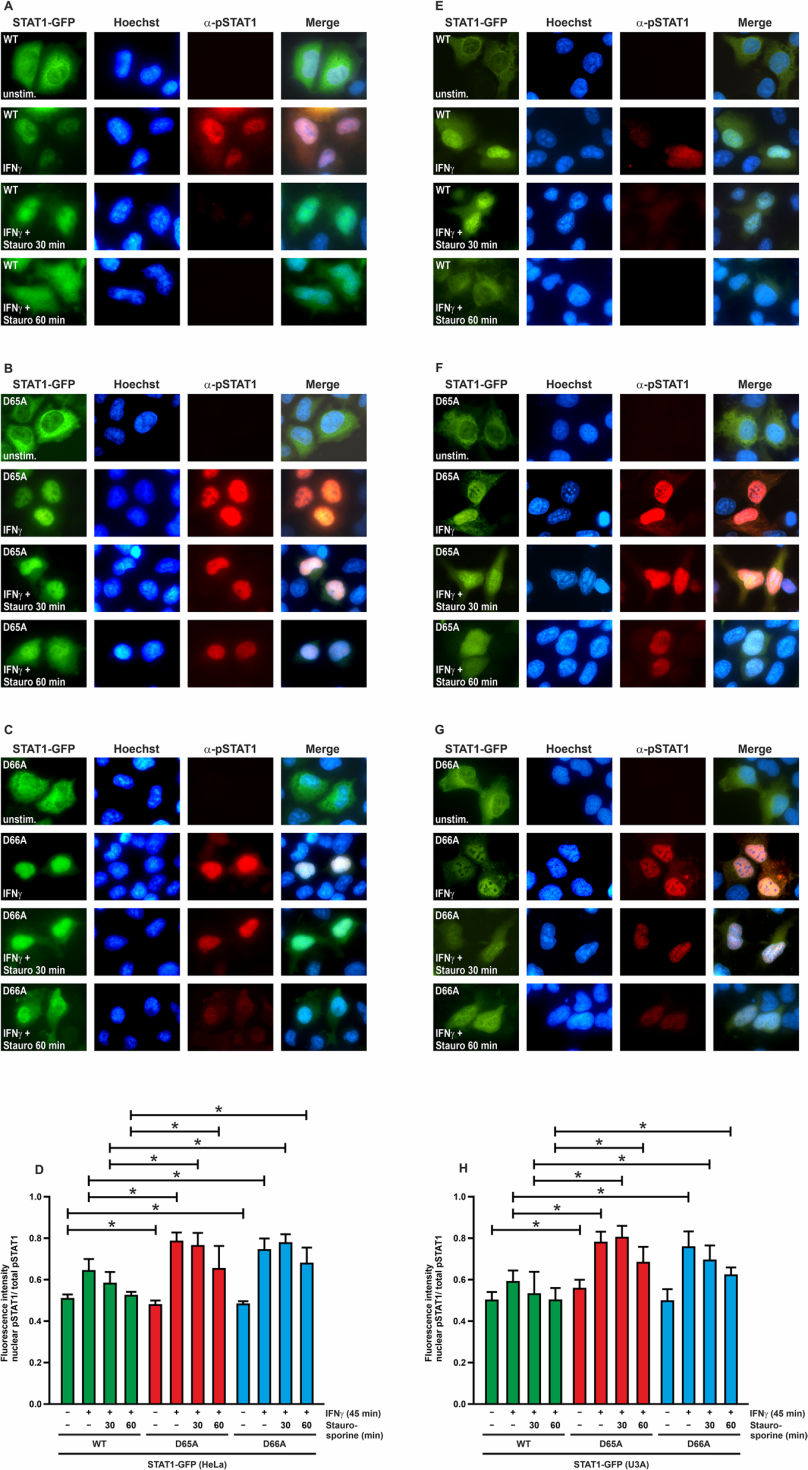
STAT1-D65A and -D66A show prolonged nuclear accumulation. **(A–D)** HeLa cells expressing GFP-tagged STAT1-WT **(A)** or the respective mutant fusion proteins **(B**,** C)** were either left untreated or exposed to 50 ng/ml of IFNγ for 45 min followed by incubation with 1 µM staurosporine for the specified times (0 min, 30 min, 60 min). Cells were immunocytochemically stained using a phosphotyrosine-specific anti-STAT1 antibody. **(D)** Quantification of immunofluorescence intensities from at least 15 cells displayed as the ratio of nuclear-to-total phosphorylated STAT1. Significant differences between the WT protein and the mutants are marked by asterisks. **(E–H)** Similar experiments to those described in **(A–D)** except that U3A cells expressing STAT1-GFP-WT **(E)** or the respective mutants -D65A **(F)** and -D66A **(G)** were used. Note that in both cell types the two aspartic acid-to-alanine mutants show a more pronounced and prolonged phase of nuclear accumulation following cytokine exposure

Immunofluorescence microscopy was employed to study the nuclear accumulation kinetics of GFP-tagged STAT1 in HeLa cells (Fig. 5A–D). Cells were either left untreated or stimulated with IFNγ for 45 min, prior to the addition of staurosporine for 0 min, 30 min, and 60 min. Immunocytochemical staining using a phosphotyrosine-specific anti-STAT1 antibody showed no specific labeling in unstimulated cells, while a prominent signal was detected in the nuclei of cytokine-stimulated cells in the absence of staurosporine. Already after 30 min of exposure to the kinase inhibitor, the fluorescent signal decreased significantly below the threshold level and was no longer detectable after an additional 30 min for the WT molecule. The two mutants similarly showed a cytokine-inducible nuclear accumulation, however, this phase was significantly extended, as phosphotyrosinylated STAT1 was present in the nuclei for up to 60 min after adding the kinase inhibitor. Similar results showing a prolonged phase of nuclear accumulation were found in U3A cells expressing GFP-tagged (Fig. 5E–H) and untagged STAT1-D65A and -D66A (Supplemental Fig. 1A–D). The hyperphosphorylation of STAT1-D65A was confirmed for Flag-tagged STAT1-D65A, which displayed a more intense nuclear immunoreactivity upon IFNγ stimulation than the WT protein (Supplemental Fig. 1E–G).

### Preserved nuclear export of phosphorylated STAT1-D65A-NES

The prolonged nuclear presence of the hyperphosphorylated D65A mutant may result from a higher affinity to genomic DNA, which protects DNA-bound STAT1 complexes from being dephosphorylated by the inactivating nuclear phosphatase. To test this hypothesis, we generated STAT1 fusion proteins with a hyperactive nuclear export signal (NES) located between the amino-terminal STAT1 position and the carboxyl-terminal GFP domain [24]. Due to its hyperactive nuclear export, tyrosine-phosphorylated STAT1-WT-NES failed to accumulate in the nucleus of IFNγ-treated cells. A lack of nuclear accumulation was also seen when the hyperactive nuclear export was inhibited by incubating the cells with 5 ng/ml of the export blocker leptomycin B (LMB). However, the combined exposure to IFNγ and LMB resulted in a restored nuclear accumulation of phosphorylated STAT1-WT-NES. A similar pattern of nuclear accumulation was also observed for the D65A-NES mutant, indicating that the phenotype of the mutant could not be explained by enhanced DNA binding. This is in contrast to E559A-NES [25], used here as a positive control, which displayed nuclear accumulation already upon exclusive treatment with IFNγ (Supplemental Fig. 1H–K).

## Discussion

Recently, Scott and colleagues described a novel STAT1 GOF mutation in female relatives (aged 5, 39, and 70 years), who, besides experiencing persistent CMC, suffered from mild to severe, long-lasting esophageal symptoms [9]. Before receiving a medical diagnosis, both women, for decades, experienced dysphagia and events of food impaction, which affected their health-related quality of life. The younger adult at age 34, finally, after various endoscopic diagnostic procedures, met the requirements of a diagnosis of eosinophilic esophagitis. Among the three first-line relatives, a genetic assessment identified a heterozygous missense mutation in the *STAT1* gene located in the N-terminal domain of the transcription factor (c.194 A > C, p.D65A). Hyperphosphorylation of this particular STAT1 mutant was demonstrated via immunoblotting, while quantitative PCR revealed increased transcription of *CXCL9* and *CXCL10* upon IFNγ stimulation. In contrast, under IFNα stimulation, only *CXCL9* exhibited a similar increase.

In the present study, we aimed to investigate in more detail the molecular phenotype of the reported D65A mutation linked to eosinophilic esophagitis using a site-directed mutagenesis approach and replaced the aspartic acid residues in position 65 and 66. Both alanine mutants showed increased STAT1 tyrosine phosphorylation accompanied by a prolonged nuclear accumulation phase. Due to their increased tyrosine phosphorylation, the DNA-binding activity in cell lysates expressing D65A and D66A mutants was higher than the WT molecule. However, their dissociation rate from a single GAS site remained unaltered compared to those of the WT protein, as determined in a competition gelshift experiment with exposure to a 750-fold excess of unlabeled GAS probe. Treatment of cells with IFNγ did not restore the defective nuclear accumulation of a fusion protein with a heterotopic NES element positioned in the STAT1-D65A construct. Therefore, the phenotype of STAT1-D65A cannot be explained as a DNA-binding mutant exhibiting a higher affinity to genomic DNA than the WT molecule. Despite its hyperphosphorylation, at a global level the D65A mutant did not upregulate transcription of all endogenous, cytokine-driven target genes significantly higher than the WT molecule such as *STAT1*, *IRF1*, *GBP1*, and *MIG*. Nevertheless, all three STAT1 variants executed a cytokine-dependent increase in gene activation. Compared to the WT protein, gene activation by the mutants was particularly enhanced for genes containing a one-and-a-half GAS motif in their promoter region. These motifs, which consist of a consensus binding site (5´-TTCNNNGAA-3´) separated by 10 bp from a half binding site (5´-TTC/GAA-3´), are located in the *MCP1* and *CXCL10* promoter region [28]. Both genes play essential roles in immune cell activation, regulation, and migration and were significantly upregulated upon cytokine induction by STAT1-D65A and -D66A.

The stability of STAT1 binding to DNA at a single GAS site is reinforced by reciprocal N-terminal interactions forming tetrameric complexes [29]. The F77 residue located within the N-terminal domain is indispensable for cooperative DNA binding mediated through tetramerization of two adjacent dimers [30, 31]. The surface-exposed side chains of the two critical aspartic acid residues D65 and D66 are located on different α-helices from D92 and E96, representing two known negatively charged residues reported to be involved in the dissociation of tyrosine-phosphorylated tetrameric complexes from DNA [32, 33]. In the crystal structure of a tetrameric complex of antiparallel aligned protomers not bound to DNA, the side chain of D65 is 4.4 Å away from the residue P252 located in the coiled-coil domain [32]. The residue D66 has an even shorter distance of 2.9 Å from its neighbor C255 in the antiparallel tetrameric conformation. These putative interactions may contribute to the formation of the non-DNA-bound antiparallel tetrameric complex, which are destabilized in the case of the aspartic acid-to-alanine mutants. In the hyperphosphorylated mutants, the equilibrium between antiparallel and parallel STAT1 dimer conformations may be shifted to the transcriptional active parallel orientation. Given that only parallel dimers can efficiently interact with DNA, thereby protecting the DNA-bound STAT1 from being a substrate for the inactivating nuclear phosphatase, a shift towards the parallel conformation results in prolonged DNA binding and thus hyperphosphorylation [34, 35]. The pathogenic D65 substitution mutation associated with EoE resides in the N-terminus of STAT1, while the known GOF mutations described so far are predominantly located on a reciprocal interaction surface formed by the coiled-coil and DNA-binding domains.

STAT1 and its homolog STAT3 have been shown to form heterodimers and, moreover, act antagonistically as homodimers with respect to their biological functions. Activated by interferons and interleukins, STAT1 plays a key role in antiviral defense, tumor suppression, and immune regulation, while exhibiting anti-proliferative and pro-apoptotic functions, promoting macrophage activation and chronic inflammation [10]. In contrast, STAT3 supports oncogenesis by inhibiting apoptosis, enhancing cell proliferation, and modulating anti-tumor immune responses through inflammatory suppression and regulatory T-cell expansion [36, 37].

Due to their antagonistic functions, a hyperactive STAT1 pathway in carriers of STAT1 GOF mutations may inhibit the signaling through STAT3, thereby mimicking the effects of STAT3 loss-of-function mutations such as hyper-IgE syndrome. The autosomal-dominant hyper-IgE syndrome is clinically characterized by elevated serum IgE levels, impaired neutrophil function, and recurrent infections such as staphylococcal abscesses, eczema, and pneumonia [38, 39]. Furthermore, patients with genetic defects in the IL-6-dependent STAT3 pathway, fundamental for T-cell differentiation (Th17/Th22), show local deficiencies in IL-17 and IL-22 production and are more likely to suffer from allergic fungal rhinosinusitis [40]. A Mendelian genetic correlation has been observed between hyper-IgE syndrome and EoE [2]. Impaired STAT3 signaling leads to dysfunctional Th17 cell differentiation, which is associated with an impaired host defense against common bacterial (*Staphylococcus aureus*) or fungal (*Candida albicans*) pathogens [41]. Additionally, patients diagnosed with hyper-IgE syndrome show an increased expression of pro-inflammatory gene transcripts [42], suggesting an overexpression of STAT1-regulated pathways. The observation that hyper-IgE syndrome caused by STAT3 loss-of-function mutations and EoE share some similar symptoms including atopic manifestations suggests that both diseases are triggered by an impaired balance between STAT1 and STAT3 activation [9]. Transgenic mice with inducible overexpression of IL-13 in esophageal epithelial cells exhibit increased epithelial proliferation that is dependent on STAT3 [43]. Using single-cell RNA sequencing data from EoE biopsy tissue, Lal and colleagues found a distinct IFN response signature in transitional epithelial cells that correlates with markers of EoE activity such as eosinophil count and endoscopic reference values [44].

In summary, our data demonstrate that the two substitution mutants with an exchange of adjacent aspartic acid residues to alanine exhibit a hyperactive transcriptional activity, which in the case of the newly characterized D66A is even higher than the pathogenic D65A mutation. Thus, we show that not only D65A, but also STAT1-D66A belongs to a novel class of GOF mutations in the N-terminal domain, which may be involved in the development of EoE. Besides the clinical evaluation of human cases, the generation of knock-in mouse models might be a suitable approach to decipher the pathogenic significance of the homologous missense mutations in an organismic context.

## Electronic supplementary material

Below is the link to the electronic supplementary material.

Supplementary Material 1

## Data Availability

All data generated or analyzed during this study are included in this published article and its Additional file 1.
