# Supplementary material for: Characterization of a pathogenic gain-of-function mutation in the N-terminal domain of STAT1 which is reported to be associated with eosinophilic esophagitis
Source: Cell Commun Signal. 2025 Aug 7;23:367. doi: 10.1186/s12964-025-02330-9 (PMC12329892; doi:10.1186/s12964-025-02330-9)
Supplement: Supplementary file 1 — Supplementary Material 1 [file 12964_2025_2330_MOESM1_ESM.pdf]

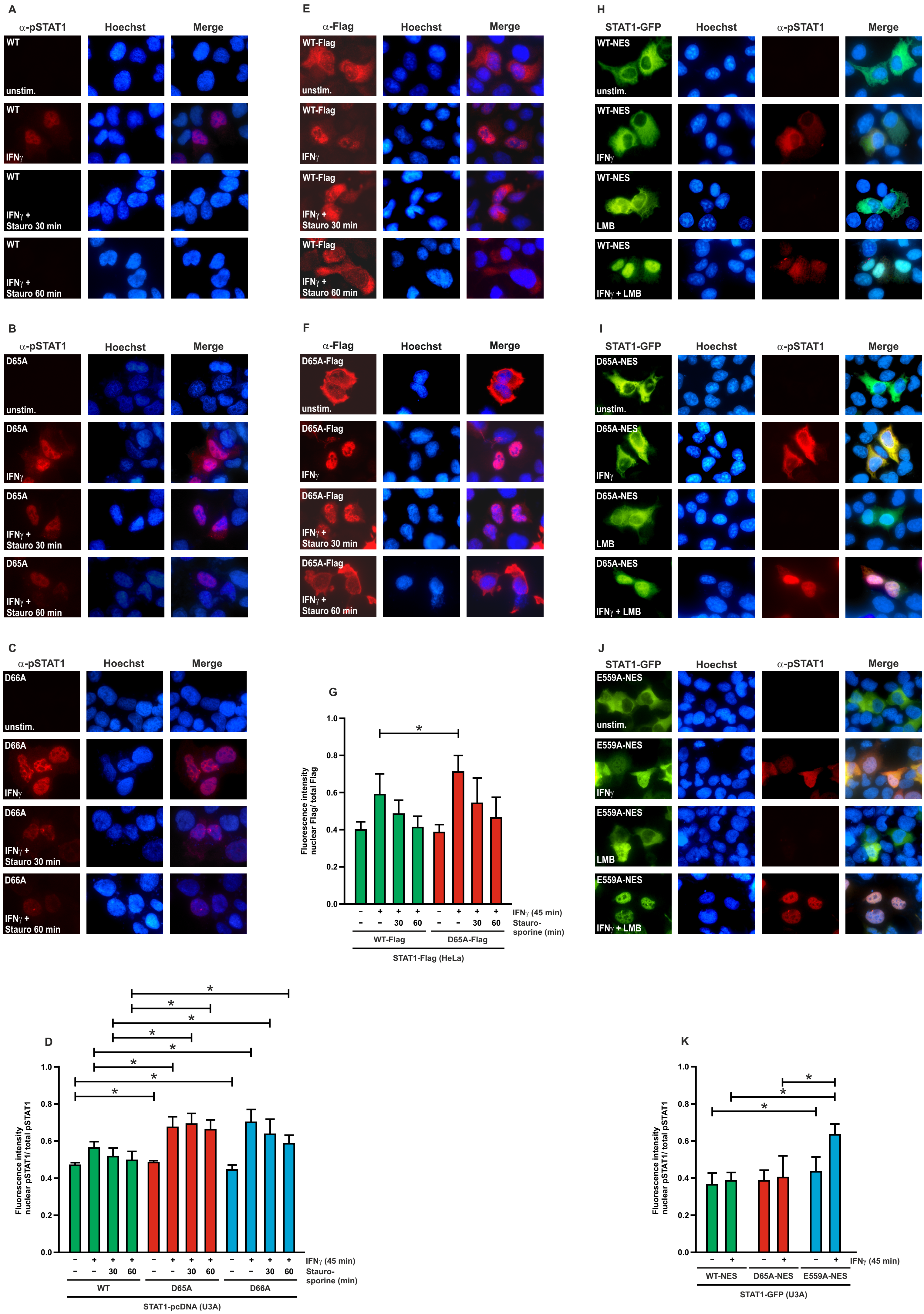

Supplemental Figure 1

Supplemental Figure 1: The elevated nuclear retention of STAT1-D65A does not result from increased DNA binding

(A–D) Prolonged tyrosine phosphorylation and nuclear accumulation of STAT1 mutants. STAT1-deficient U3A cells expressing untagged WT **(A)** or the respective mutants D65A **(B)** and D66A **(C)** were either left untreated or exposed to 50 ng/ml of IFNγ for 45 min followed by incubation with 1 µM staurosporine for the indicated times. Cells were immunocytochemically stained using a phosphotyrosine-specific anti-STAT1 antibody. **(D)** Quantification of the results from **(A–C)** showing immunofluorescence intensity data as the ratio of nuclear-to-total phosphorylated STAT1 from at least 15 cells. Significant differences between the WT protein and the respective mutants are marked with asterisks. **(E–G)** Elevated nuclear accumulation of Flag-tagged STAT1-D65A. HeLa cells expressing Flag-tagged WT **(E)** or mutant STAT1 **(F)** were either left untreated or exposed to 50 ng/ml of IFNγ for 45 min, followed by incubation with 1 µM staurosporine and subsequent staining of the fixed cells with an anti-Flag antibody. **(G)** Quantification of the nuclear-to-total anti-Flag immunoreactivity from at least 15 cells in each sample **(E, F)**. **(H–K)** D65A-NES exhibit normal DNA-binding affinity. U3A cells were transfected with plasmids coding for WT **(H)** or mutant **(I, J)** STAT1-GFP fusion proteins with a STAT1-derived nuclear export signal (NES). STAT1-E559A-GFP-NES **(J)** served as a positive control. Transfected cells were either left untreated or exposed to IFNγ (50 ng/ml), leptomycin B (LMB, 5 ng/ml) or the combination of both for 45 min each. Cells were immunocytochemically stained for phospho-STAT1. **(K)** The quantification shows the mean immunofluorescence intensity data given as nuclear-to-total phosphorylated STAT1 for the indicated mutants in untreated and cytokine-treated cells.
